# Ligustrazine Injection for Chronic Pulmonary Heart Disease: A Systematic Review and Meta-Analysis

**DOI:** 10.1155/2012/792726

**Published:** 2011-08-11

**Authors:** Li Jian-sheng, Wang Hai-feng, Bai Yun-ping, Li Su-yun, Yu Xue-qing, Li Ya

**Affiliations:** ^1^Department of Respiratory Medicine, The First Affiliated Hospital of Henan University of Traditional Chinese Medicine, Zhengzhou 450000, China; ^2^The Geriatric Department of Henan University of Traditional Chinese Medicine, Zhengzhou 450008, China

## Abstract

*Objective*. This study was intended to evaluate the efficacy and safety of ligustrazine injection for chronic pulmonary heart disease (CPHD). *Method*. Randomized controlled trials (RCTs) of clinical therapeutic studies on CPHD when using ligustrazine injection were included. Searches were applied to the following electronic databases: the PubMed, the Cochrane Library, EMBASE, CBM, and AMED. No language restriction was used. All trials included were analyzed according to the criteria of the Cochrane Handbook. Review Manager 5.0 software was used for data analysis. *Result*. 34 RCTs with low methodological quality were included. Compared to conventional medicine treatment alone, ligustrazine injection plus conventional medicine treatment showed improvement in New York Heart Association classification of clinical status (Odds ratio 0.22; 95% CI 0.17 to 0.28) and depression of pulmonary artery hypertension (weighted mean difference −4.77; 95% CI −5.85 to −3.68). Three studies had reported adverse events. No serious adverse effects were reported. *Conclusion*. While there is some evidence that suggests potential effectiveness of ligustrazine injection for CPHD, the results were limited by the methodological flaws of the studies. High quality studies are needed to provide clear evidence for the future use of ligustrazine injection.

## 1. Introduction

Chronic pulmonary heart disease (CPHD) is a rising major public health problem worldwide [1]. In China, many traditional Chinese patent medicines (TCPMs) are regularly used in CPHD patients in hospital. Few studies reporting the effectiveness and safety of many commonly used TCPMs for cor pulmonale have been published in English. Sichuan lovage rhizome is one kind of traditional Chinese herbal medicines, which has the function of promoting blood circulation and qi flowing, dispelling pathogenic wind and relieving pain. It has long been used for the treatment of heart disease and cerebral disease in China. Ligustrazine is an isolated alkali extracted from Sichuan lovage rhizome. It is the formula is 2,3,5,6-tetramethyl pyrazine. It has effect of dilating blood vessels, inhibiting platelet aggregation and preventing thrombopoiesis. There are two common types: ligustrazine phosphate and ligustrazine hydrochloride. Several clinical trials have shown that ligustrazine injection might have the therapeutic effect of improving cardiac function in patients with CPHD. However, the quality of these trials has not been assessed systematically. Furthermore, the spontaneous evolution of CPHD is difficult to predict. A systematic paper will be beneficial for current practice and directive for continuing research for new treatment regimens. The objective of this review was to assess both positive and negative effects of ligustrazine injection plus conventional medicine treatment versus conventional medicine treatment in all types of CPHD in adults. 

## 2. Materials and Methods

### 2.1. Eligibility Criteria

The eligibility criteria for conducting the survey included administration of ligustrazine hydrochloride injection for the treatment of CPHD and duration of treatment and also included trials involving patients of any age, sex, or CPHD stage. However, we excluded pharmacokinetic studies, nonrandomized evaluations, and animal/laboratory studies.

### 2.2. Search Strategy

Two reviewers (L. S. Y. and W. Z. W.) identified relevant randomised controlled trials independently, including all languages, by a systematic search of PubMed (1990–2010), Chinese Biomedical Database (CDROM, 1990–2010), EMBASE (1990 to December 2010), AMED (1990 to December 2010), Chinese BioMedical Literature Database (CBM, 1990 to December 2010), the Cochrane Library (Issue 2, 2010), and BIOSIS (1997 to 2008). With the following terms: (cor pulmonale or chronic pulmonary heart disease) AND (Ligustrazine hydrochloride injection or ligustrazine hydrochloride injection). We used the wild card term “∗” to enhance the sensitivity of our search strategy. Reviewers scanned the bibliographies of all retrieved trials and other relevant publications, including reviews and meta-analyses, to ensure a thorough search.

### 2.3. Study Selection and Data Extraction

Two reviewers (L. S. Y. and L. Y.) extracted data and evaluated data's quality and content independently. We conducted data extraction using a standardized procedure. Initially, abstracts were screened to exclude obviously ineligible reports, and then all remaining articles were reviewed. We classified trials and abstracts according to drug, patient characteristics, study design, and therapy duration. Reviewing study design included the following criteria: methods of sequence generation, allocation concealment, complete description of those who were blinded [2], and use of intention-to-treat analysis and whether the trial was stopped prior to the planned duration, all methodological features in addition capable of impacting effect sizes.

The primary outcome measures included (1) New York Heart Association classification (NYHA) of clinical status; (2) adverse events. The secondary outcome measures included (1) death, (2) hemodynamics, (3) quality of life as measured by various instruments.

The data was entered into an electronic database by the two reviewers separately, avoiding duplicate entries; in the case where the two entries did not match, an inspection will be conducted, and a third person may be involved for verification. In order to obtain full information regarding conference abstracts, we had contacted the study authors by email and/or telephone communication.

### 2.4. Data Analysis

The statistical package (RevMan 5.0) provided by the Cochrane Collaboration was used to analyze the data. Dichotomous data was presented as Odds Ratio (OR), with 95% confidence intervals (CIs). Continuous outcomes were presented as weighted mean difference, with 95% CI. Analyses were performed by intention-to-treat where possible. Otherwise, for dichotomous outcomes, patients with incomplete or missing data were included in sensitivity analyses by counting them alternately as treatment failures or successes. For continuous variables we used simple sensitivity analysis to test how robust the results were under different assumptions about what might have happened to patients with missing data. 

Meta-analysis was only performed within comparisons where individual trials were compared with similar treatment and control interventions.

## 3. Results

### 3.1. Study Identification and Characteristics

**Figure 1:**
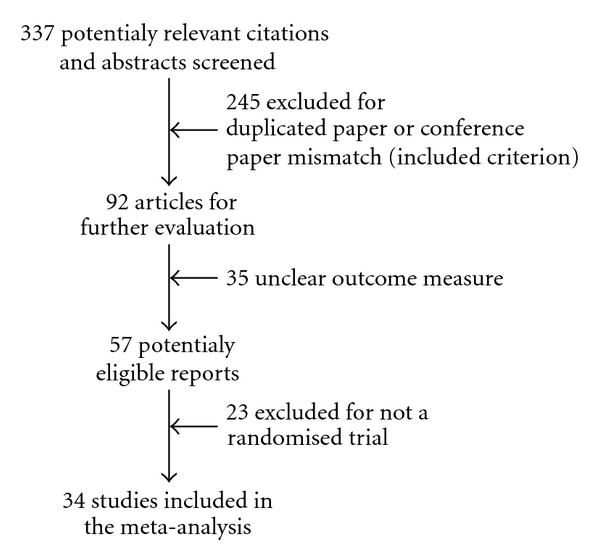
The Quality of Reporting of Meta-analyses flow diagram for the meta-analysis.

An initial search identified 337 potentially relevant articles. A total of 245 articles were initially excluded because of duplicate publication, and then 35 articles were excluded because they did not meet our inclusion criteria. From the identified 57 potentially eligible reports, 23 articles out of 57 were excluded for further assessment. The most frequent reasons for exclusion were other types of intervention examined, either no clinical outcomes reported, or not a randomized trial so that a total of 34 studies (2319 participants) were included into the review. All studies were published in Chinese.  Figure 1 summarized the search results based on the quality of reporting of meta-analyses flow diagram. The bibliographic details of these trials are given in Table 1.

The age of patients in the included studies ranged from 37 to 85 years old. Most trials included more males (54% to 89%) than females; only 1 trial included more females (55%). All included trials applied standard western medicine diagnostic criteria for CPHD, although some trials also used TCM criteria in addition. The dose range of ligustrazine hydrochloride injection was from 60 mg to 800 mg and dose route was intravenous injection in all included studies. The total duration of treatment varied from 7 to 15 days.

None of the trials have undergone assessment of patients' routine living ability after treatment; some trials had measurement of blood gas analysis and hemodynamic parameters.

Only ligustrazine injection was studied in these included trials. None of the trials was randomized, double-blind, placebo controlled. All included trials were designed to compare ligustrazine hydrochloride injection plus conventional treatment with the conventional treatment alone.

#### 3.1.1. Study of Quality

The methodological quality of most included trials was generally “poor.” No trials reported using randomization method, yet only 1 trial mentioned double-blind [3]; none of the trials was properly randomized, double blinded, placebo controlled. None of the trials had grade a level of adequate concealment of randomization. The remaining 33 possible RCTs did not describe the way of randomization. None of the trials blinded the assessment of outcome. None of the trials reported the number of patients that were lost to followup and whether they had used intention-to-treat analysis.

### 3.2. Publication Bias

Although we conducted comprehensive searches and tried to avoid bias, since all trials were published in Chinese, we could not exclude potential publication bias.

### 3.3. Primary Outcomes

#### 3.3.1. New York Heart Association Classification of Clinical Status (NYHA Class Improved <1 Class)

**Figure 2:**
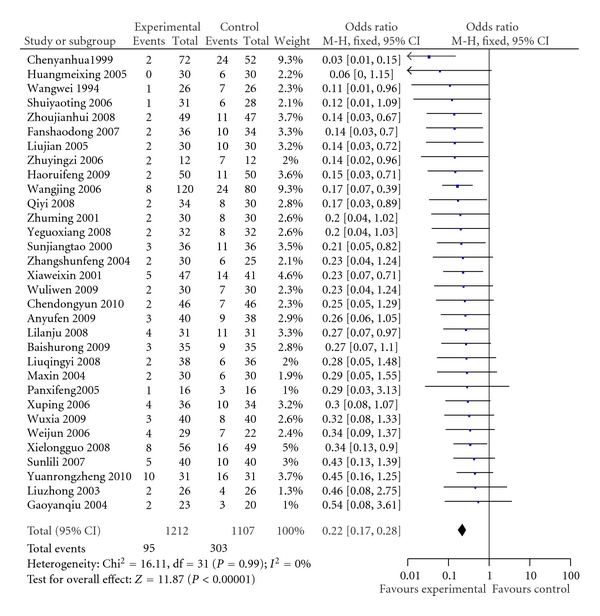
Comparison of ligustrazine hydrochloride injection + conventional treatment versus conventional treatment. Lack of improvement in heart failure (NYHA class improved <I class or worsening of heart failure). M-H: Mantel-Haenszel formula.

32 trials (2319 patients) concluded an improvement in heart failure [3–34]. The entire heart function classification system used the NYHA functional classification method. A meta-analysis of data on change of NYHA functional classification showed that ligustrazine injection plus conventional treatment had more benefit compared with conventional treatment alone. The summary odds ratio (OR) of NYHA class improved <I class, or worsening of heart failure was 0.22 (OR, 0.22; 95% CI, 0.17 to 0.28)  (Figure 2).

**Adverse Events**

**The duration of treatment in all included studies was short (7 to 15 Days). Outcomes were measured at the End of treatment**

No obvious adverse events occurred in 21 trials, and no adverse events were reported in 11 trials. One trial reported adverse events [17], and the adverse events were described as dry mouth and mild drowsiness, but they were not severe. It will recover without special treatment. There were no adverse events in control group. Although we did not find any related information about adverse effects recorded, potential or long-time adverse effects in the included trials could not be excluded.

### 3.4. Secondary Outcomes

#### 3.4.1. Death

**Figure 3:**
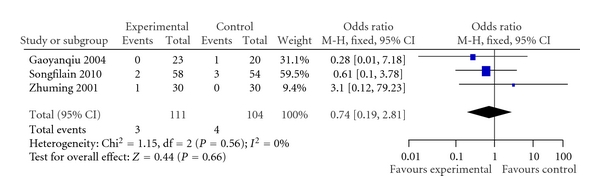
Comparison of death rate in ligustrazine hydrochloride injection + conventional treatment versus conventional treatment.

Deaths were reported in only 3 trials [27, 32, 35]. The total duration of treatment varied from 10 to 15 days. No death cases reported in the remaining trials. There was no statistically significant difference between 2 groups (OR, 0.74; 95% CI, 0.19 to 2.81)  (Figure 3).

#### 3.4.2. Hemodynamics

Two trials provided data for Doppler echocardiography hemodynamic change [11, 22]. At the end of treatment, one trial showed increased ejection fraction (weighted mean difference (WMD) 0.09, 95% CI 0.07 to 0.11)  [11, 22] of ligustrazine injection plus conventional medicine treatment group. One trial showed a significant increase in cardiac output (mean of the experimental group and control group was 4.46 L/min to 3.07 L/min), increased ejection fraction (mean of the experimental group and control group was 71% to 56.9%) and increased stroke volume (mean of the experimental group and control group is 56.8 mL to 36.8 mL) [11], and there was significant difference between the two groups, but unfortunately the trial did not provide proper statistic analysis (no standard deviation).

**Figure 4:**
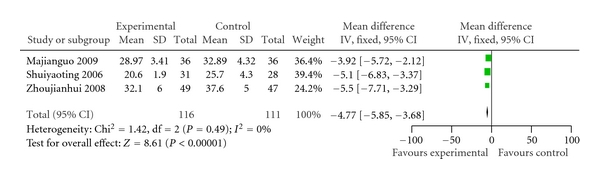
Comparison of mean pulmonary arterial pressure in ligustrazine hydrochloride injection + conventional treatment versus conventional treatment. SD: standard deviation.

Three trials showed a significant decrease mean pulmonary arterial pressure (WMD −4.77, 95% CI −5.85 to −3.68) [11, 19, 36]  (Figure 4).

#### 3.4.3. Quality of Life

No trial assessed quality of life.

## 4. Discussion

In many years, western medicine has made great progress and had become the dominating and leading medical treatment research and development worldwide. However, it has been increasingly recognized that western medicine may sometimes fail to treat any illness, whereas such illness is reportedly improved by the so-called complementary and alternative medicine based on a different theory. Few relevant articles on TCM for CPHD have been published in the English medical journals, and the limited evaluation of TCM beyond China reduces its external validity.

This review of randomized trials shows the current evidence in ligustrazine injection for CPHD. Treatment was applied according to a diagnosis of CPHD without further syndrome differentiation according to traditional Chinese methodology. Study sizes were generally insufficient. All conclusions, therefore, were impropriate. There were no descriptions of allocation concealment and no assurances that blinding was maintained. The type of conventional medicine used and the dosages were often impropriate reported, so it was not certain that all studies used similar medications.

Ligustrazine injection plus conventional medicine treatment showed significantly less outcomes of decreased heart function as with “no change or worse” in comparison with the treatment of conventional medicine alone. With measurement using NYHA of clinical status (OR, 0.22; 95% CI, 0.17 to 0.28), the treatment group had, on average, 22 percent less than those treated with conventional medicine alone. These results were positively encouraging and promising of combining ligustrazine injection with conventional treatment, which might be beneficial to heart function. The positive findings were also observed in mean pulmonary arterial pressure outcome (WMD −4.77, 95% CI −5.85 to −3.68). In our opinion, such an average difference would be noticeable and clinically meaningful. In addition, patients treated with ligustrazine injection may have a beneficial effect on cardiac output, ejection fraction, and stroke volume, however, there is just one study providing this result. As for other outcomes, such as death, there were just 3 trials (total *n* = 215), which showed no significant difference in favour of the ligustrazine injection plus conventional combination treatment (OR, 0.74; 95% CI, 0.19 to 2.81).

Adverse effects were reported by Shaodong and Xuefen (2007) [17] as only dry mouth and mild drowsiness, and the adverse events were not severe. They spontaneously recovered without special treatment. No obvious adverse events occurred in 21 trials. 11 trials did not report adverse effects and these were not significantly different between the two treatments. However, the concrete conclusion regarding safety cannot be determined from this review due to the limited evidence provided by the eligible trials. In order to proper assess the safety of ligustrazine injection, large-scale clinical trials with long-term followup are required.

The application of traditional Chinese herbal medicine is fundamentally prescribed with syndrome differentiation. Failure to apply syndrome differentiation may result in treatments being ineffective or even harmful. Despite this, there is some evidence that these Chinese herbal medicines, combined with conventional medicine and given in a way that is not in keeping with their normal use within traditional Chinese medicine, may be beneficial for people with CPHD across a range of outcomes. If these medicines are used properly the positive effects could be enhanced. 

The improvement of heart failure was noticed in the survey; however, the effects may not be true due to improper study design or method used, such as lack of randomization or allocation concealment. Publication bias could also be a factor. we tried to take all measures to contact authors to get further information either by telephone, letter, or email. Unfortunately, we got no replies, and we are not sure, the trials were conducted as true RCT. Subsequently, no clear conclusion could be made from these trials.

## 5. Conclusions

In conclusion, because of the unclear methodological quality of these identified trials, a definite conclusion on efficacy and adverse events associated with ligustrazine injection cannot be drawn from this review. We found no evidence of a beneficial effect on the primary measure of efficacy to support the routine use of ligustrazine injection for CPHD. However the drug appeared to have no major adverse effects, and if its positive effects were confirmed by comprehensive clinical trials, it would lead to many promising treatments for CPHD and could benefit all patients all over the world. Therefore, further thorough investigation, large-scale, proper study design, randomized trials of ligustrazine injection for CPHD will be required to justify the effects reported here. Future trials should overcome the limitations of the trials presented in this review; particularly, they should assure adequate concealment of allocation and blinding of outcome assessors and use functional outcome as the primary outcome measured at long-term followup. Reports of the trials should conform to the recommendations of the CONSORT statement. If reliable RCT results confirmed ligustrazine injection's positive effects for treatment of CPHD, it would be blessing news for CPHD patients worldwide.

## Figures and Tables

**Table 1:** Characteristics of included studies.

Included studies		Methods		Participants			Interventions		Outcomes				
Date of study	First author	Double-blind	Settings	*n*	Age (years)	Gender	Experiment group	Control group	Death	Clinical status	Quality of life	Hemorheology	Adverse effect
1999	Chen	Yes	H	72/52	45–85	M, F	Conventional medicine treatment plus ligustrazine injection 200–240 mg qd. 14 days	Conventional medicine treatment	NK	Yes	No	No	NK

2010	Chen	No	H	46/46	61–77 69 ± 5.6	M, F	Conventional medicine treatment plus ligustrazine injection 100 mg qd. 14 days	Conventional medicine treatment	NK	Yes	No	No	NK

2010	Yuan	No	H	31/31	NK	M, F	Conventional medicine treatment plus Ligustrazine Hydrochloride injection 40 mL, 60 mL qd. 14 days	Conventional medicine treatment	No	Yes	No	Yes	NK

2009	Bai	No	H	35/35	58–80	M, F	Conventional medicine treatment plus ligustrazine injection 80 mg qd. 14 days	Conventional medicine treatment	No	Yes	No	Yes	No

2009	An	No	H	40/38	59–81	M, F	Conventional medicine treatment plus ligustrazine injection 120 mg qd. 14 days	Conventional medicine treatment	NK	Yes	No	No	NK

2009	Hao	No	H	50/50	55–75	M, F	Conventional medicine treatment plus ligustrazine injection 400 mg qd. 10 days	Conventional medicine treatment	NK	Yes	No	No	NK

2009	Wu	No	H	30/30	62 ± 10.5/60 ± 11.5	M, F	Conventional medicine treatment plus ligustrazine injection 400 mg qd. 10 days	Conventional medicine treatment	NK	Yes	No	No	NK

2009	Wu	No	H	40/40	50–70	M, F	Conventional medicine treatment plus ligustrazine injection 800 mg qd. 7 days	Conventional medicine treatment	NK	No	No	No	NK

2009	Ma	No	H	36/36	48–82	M, F	Conventional medicine treatment plus ligustrazine injection 400 mg qd. 14 days	Conventional medicine treatment	NK	Yes	No	No	NK

2008	Li	No	H	31/30	51 ± 6	M, F	Conventional medicine treatment plus ligustrazine injection 100 mg qd. 10 days	Conventional medicine treatment	NK	Yes	No	No	NK

2008	Liu	No	H	38/36	Mean 65.2/65.6	M, F	Conventional medicine treatment plus ligustrazine injection 120–240 mg qd. 10 days	Conventional medicine treatment	NK	Yes	No	Yes	NK

2008	Qi	No	H	34/30	57–82	M, F	Conventional medicine treatment plus ligustrazine injection 100 mL. qd 14 days	Conventional medicine treatment	No	Yes	No	Yes	NK

2008	Ye	No	H	32/32	65 ± 8.2/64 ± 8.6	M, F	Conventional medicine treatment plus ligustrazine injection 120 mg. qd 10 days	Conventional medicine treatment	No	Yes	No	No	No

2008	Xie	No	H	56/49	36–75 Mean 66.5/38–77 Mean 67	M, F	Conventional medicine treatment plus ligustrazine injection 80 mg. qd or bid. 10–14 days	Conventional medicine treatment	NK	Yes	No	Yes	NK

2007	Fan	No	H	22/21	72 ± 9.7/70.5 ± 8.5	M, F	Conventional medicine treatment plus Ligustrazine Hydrochloride injection 100 mL. qd. 14 days	Conventional medicine treatment	No	Yes	No	Yes	Yes

2007	Sun	No	H	40/40	63.67 ± 14.15/67.25 ± 10.64	M, F	Conventional medicine treatment plus Ligustrazine Hydrochloride injection 160 mg. qd. 14 days	Conventional medicine treatment	NK	Yes	No	Yes	NK

2006	Shui	No	H	31/28	67 ± 12/66 ± 11	M, F	Conventional medicine treatment plus ligustrazine injection 400 mg. qd. 7 days	Conventional medicine treatment	NK	Yes	No	Yes	NK

2006	Wei	No	H	29/22	59–81	M, F	Conventional medicine treatment plus ligustrazine injection 80 mg. qd. 7 days	Conventional medicine treatment	NK	Yes	No	No	NK

2006	Zhu	No	Out patient	12/12	47–85	M, F	Conventional medicine treatment plus ligustrazine injection 80 mg. qd. 10 days	Conventional medicine treatment	NK	Yes	No	No	No

2006	Wang	No	H	120/80	40–85	M, F	Conventional medicine treatment plus ligustrazine injection 100 mg. qd. 10–15 days	Conventional medicine treatment	NK	Yes	No	Yes	NK

2006	Xu	No	H	36/34	56–82, 65.4 ± 6.3	M, F	Conventional medicine treatment plus ligustrazine injection 160 mg. qd. 10 days	Conventional medicine treatment	NK	Yes	No	No	NK

2005	Huang	No	H	30/30	49–71	M, F	Conventional medicine treatment plus ligustrazine injection 80 mg. qd. 10 days	Conventional medicine treatment	Nk	Yes	No	No	Nk

2005	Liu	No	H	30/30	59–75, 64.2 ± 10.5	M, F	Conventional medicine treatment plus ligustrazine injection 400 mg. qd. 10–14 days	Conventional medicine treatment	NK	Yes	No	Yes	No

2005	Pan	No	H	16/16	60–89	M, F	Conventional medicine treatment plus ligustrazine injection 160 mg. qd. 14 days	Conventional medicine treatment	NK	Yes	No	Yes	NK

2004	Gao	No	H	23/20	55–80	M, F	Conventional medicine treatment plus ligustrazine injection 80 mg. qd. 15 days	Conventional medicine treatment	Yes	Yes	No	No	No

2004	Ma	No	H	30/30	50–70	M, F	Conventional medicine treatment plus ligustrazine injection 800 mg. qd. 7 days	Conventional medicine treatment	No	Yes	No	No	NK

2004	Zhang	No	H	30/25	53–79	M, F	Conventional medicine treatment plus ligustrazine injection 200 mg. qd. 10 days	Conventional medicine treatment	No	Yes	No	No	NK

2003	Liu	No	H	26/26	56–82	M, F	Conventional medicine treatment plus ligustrazine injection 200 mg. qd. 14 days	Conventional medicine treatment	No	Yes	No	Yes	NO

2001	Xia	No	H	47/41	58–73	M, F	Conventional medicine treatment plus ligustrazine injection 200–240 mg. qd. 14 days	Conventional medicine treatment	NK	Yes	No	No	No

2001	Zhu	No	H	30/30	stratified	M, F	Conventional medicine treatment plus ligustrazine injection 800 mg. qd. 10 days	Conventional medicine treatment	Yes	Yes	No	Yes	No

2000	Sun	No	H	36/36	NK	M, F	Conventional medicine treatment plus ligustrazine injection 400 mg. qd. 14 days	Conventional medicine treatment	No	Yes	No	No	NK

1994	Wang	No	H	26/26	37–77	M, F	Conventional medicine treatment plus ligustrazine injection 160 mg. qd. 10 days	Conventional medicine treatment	NK	No	No	No	No

2008	Zhou	No	H	49/47	NK	M, F	Conventional medicine treatment plus ligustrazine injection 80 mg. qd. 10 days	Conventional medicine treatment	NK	Yes	No	No	No

H: hospital; M: male; F: female; NK: not known.
